# 521. Predictors of Hospitalization Due to Coronavirus Disease 2019 (COVID-19) at a Veterans Affairs Medical Center

**DOI:** 10.1093/ofid/ofab466.720

**Published:** 2021-12-04

**Authors:** Macy Ho, Sarah Le, Rajkumar J Sevak, Jamie G Chapman-Bueno, Stephen M Berman, Patricia Chun, Yong S Moon

**Affiliations:** 1 VA Long Beach Healthcare System, Manhattan Beach, CA; 2 VA Long Beach HCS, Long Beach, California; 3 University of the Pacific School of Pharmacy, Stockton, California; 4 University of the Pacific School of Pharmacy/VA Long Beach Healthcare System, Long Beach, California

## Abstract

**Background:**

During the COVID-19 pandemic, the Veterans Affairs Long Beach Healthcare System (VALB HCS) saw a surge of patients with a positive SARS-CoV-2 test. There is a lack of guidance on triaging patients with COVID-19 in the clinical literature. To address this need, our study evaluated factors that predicted hospitalization of patients due to COVID-19.

**Methods:**

This was a retrospective cohort study of patients with a positive SARS-CoV-2 test and medical evaluation at the VALB HCS between August 1 and December 31, 2020. SARS-CoV-2 positive patients admitted to the hospital for a non-COVID-19 related diagnosis were excluded. At the time of initial evaluation, demographic, clinical, and laboratory data, and PCR cycle threshold were collected and compared between patients admitted to the hospital and those managed in the community. A multiple logistic regression analysis was conducted to evaluate predictors for hospitalization due to COVID-19.

**Results:**

Of 748 patients, 94 were admitted to the hospital and 654 were community-managed. The outcomes from the logistic regression analysis indicated that the model explained 58.8% of variance and was a significant predictor of hospitalization (X2 [8, 737] = 277.5, p< 0.0001). Patients with self-reported shortness of breath (OR=12.14, 95% CI=6.43-22.92) or diarrhea (OR=2.78, 95% CI=1.33-5.84) were more likely to be hospitalized, whereas patients with sore throat (OR=0.095, 95% CI=0.017-0.53) or body ache (OR=0.42, 95% CI=0.20-0.89) were less likely to be hospitalized than patients not having such symptoms. Every unit increase in patients’ age and temperature increased the likelihood of hospitalization by 7.6% and 62.7%, respectively. Every unit increase in patients’ diastolic pressure and SpO_2_% decreased the likelihood of hospitalization by 6.1% and 3.6%, respectively.

**Conclusion:**

Our findings indicate that patients with shortness of breath, diarrhea, temperature, and old age were more likely to be hospitalized due to COVID-19. The results may help providers in clinical decision making regarding whether to admit the patient or not. These findings may be especially helpful when hospital bed availability is limited.

**Disclosures:**

**All Authors**: No reported disclosures

